# Generation of functional liver sinusoidal endothelial-like cells from human bone marrow-derived mesenchymal stem cells

**DOI:** 10.1016/j.reth.2023.07.006

**Published:** 2023-08-01

**Authors:** Seiji Mitani, Yu Onodera, Chihiro Hosoda, Yoko Takabayashi, Asuka Sakata, Midori Shima, Kohei Tatsumi

**Affiliations:** a Advanced Medical Science of Thrombosis and Hemostasis, Nara Medical University, Kashihara, Nara 634-8521, Japan; b Medicinal Biology of Thrombosis and Hemostasis, Nara Medical University, Kashihara, Nara 634-8521, Japan

**Keywords:** Liver sinusoidal endothelial cells, Mesenchymal stem cells, Differentiation method, Liver disease

## Abstract

**Introduction:**

Liver sinusoidal endothelial cells (LSECs) are specialized vascular endothelial cells that play an important role in the maintenance of biological homeostasis. However, the lack of versatile human LSECs has hindered research on LSECs and development of medical technologies for liver diseases including hemophilia A. In this study, we developed a technique to induce LSEC differentiation from human bone marrow-derived mesenchymal stem cells (BM-MSCs).

**Methods:**

To induce LSECs from human BM-MSCs, cytokines and chemical compounds associated with signaling implicated in LSEC differentiation and liver development were screened. Then LSEC-related genes and proteins expression in the differentiated cells were analyzed by qPCR and flow cytometry analysis, respectively. LSEC-related functions of the differentiated cells were also examined.

**Results:**

We found that the gene expression of LSEC markers, such as *LYVE1*, was considerably increased by culturing human BM-MSCs with bone morphogenetic protein 4, fibroblast growth factor 8b, transforming growth factor-β signal inhibitor, and cyclic AMP. Furthermore, the differentiated cells expressed LSEC marker proteins and clearly demonstrated LSEC-specific functions, such as the uptake of hyaluronic acid.

**Conclusions:**

Our result indicate that the functional LSEC-like cells were successfully generated from human BM-MSCs using our established protocol.

## Highlights

• BMP4, FGF8b, TGF-β inhibitor, and cAMP treatment induce LSEC differentiation from MSC.
• MSC-derived cells express liver sinusoidal endothelial cell markers including LYVE1.
• MSC-derived cells display hyaluronan-uptake capacity, a function specific to LSEC.

## 1 Introduction

Liver sinusoidal endothelial cells (LSECs) are endothelial cells (ECs) that form microvessels in the liver. They are known to be morphologically and functionally unique vascular ECs. For example, LSECs possess a high endocytic capacity and play an important role in the clearance of waste and foreign substances from the blood, thereby maintaining homeostasis in the body [1]. Furthermore, LSECs have been reported to play important roles in the progression and suppression of liver diseases such as dyslipidemia [2], alcoholic liver disease [3], liver fibrosis [4], and liver cancer [5]. LSECs are also vital for liver regeneration [6]. In addition, LSECs have an important role in the production of coagulation factor VIII (FVIII) [7].

The deficiency of FVIII causes decreased hemostatic capacity (hemophilia A), and LSEC transplantation has therapeutic potential against hemophilia A [8,9]. However, because of the shortage of donors of human primary LSECs and because cultured LSECs have difficulty maintaining their specificity in vitro [10,11], hemophilia A treatment with human primary LSECs might be challenging. To overcome this problem, stem cell technology is anticipated as a promising approach. In various fields of stem cell research, mesenchymal stem cells (MSCs), induced pluripotent stem cells (iPSCs), and embryonic stem cells (ESCs) are used. Especially if functional LSECs can be simply and rapidly induced from proliferative human MSCs, they will be a useful cell source for transplantation because MSCs have some advantages over iPSCs and ESCs, such as a low risk of tumorigenesis. However, the reported method of differentiation of MSCs into LSECs is time-consuming (28 days) and inefficient (differentiation efficiency is < 20%), and the functions associated with LSECs of their derivatives are unclear [12]. Therefore, developing a simpler and more efficient protocol for differentiating MSCs into functional LSECs is important.

In this study, we developed a method to induce the differentiation of human bone marrow-derived MSCs (BM-MSCs) into functional LSECs. First, we screened cytokines and small-molecule compounds for differentiation, utilizing existing knowledge of liver development and LSEC differentiation. Subsequently, we evaluated the gene and protein expression levels in the cells differentiated from human BM-MSCs and their LSEC-specific functions. Our study shows that the combination of bone morphogenetic protein (BMP) 4, fibroblast growth factor (FGF) 8b, transforming growth factor-β (TGF-β) signal inhibitor, and cyclic AMP (cAMP) can induce efficient differentiation of human BM-MSCs into functional LSEC-like cells.

## 2 Materials and methods

### 2.1 Materials

Total RNA was extracted from human hepatic sinusoidal endothelial cell pellets (#CP5000; ScienCell, Carlsbad, CA) and reverse transcribed to cDNA (hLSEC#2). Total RNA of human adult liver tissue (pool of five donors) (R1234149-P; BioChain, Newark, CA) was reverse transcribed to cDNA.

### 2.2 Cell culture

UE7T-13 (immortalized human BM-MSCs; JCRB1154), and UCB408E7-TERT34 (immortalized human umbilical cord blood-derived MSCs [UCB-MSCs]; JCRB1546) were purchased from JCRB Cell Bank. UE7T-13 and UCB408E7-TERT34 were cultured in low glucose-Dulbecco's Modified Eagle's Medium (SIGMA-Aldrich, St. Louis, MO) supplemented with 10% FBS (fetal bovine serum), 100 U/mL penicillin, and 100 μg/mL streptomycin. HUVECs (Lonza, Basel, Switzerland) were cultured in EGM2 medium (Lonza). Human hepatic sinusoidal endothelial cells (P10652; Innoprot, Bizkaia, Spain) (hLSEC#1) were cultured in endothelial cell medium (P60104; Innoprot). The primary human bone marrow-derived MSCs used in this study were purchased from the PromoCell GmbH (Heidelberg, Germany). Primary human bone marrow-derived MSCs (C-12974; PromoCell) were cultured in Mesenchymal Stem Cell Growth Medium 2 (C-28009; PromoCell). The characteristics as MSCs including adipogenesis, chondrogenesis, and osteogenesis potencies of the primary human bone marrow-derived MSCs were checked by the supplier.

### 2.3 Differentiation of human MSCs into LSEC-like cells

The cells were dissociated into single cells and plated on a fibronectin (Fujifilm, Osaka, Japan)-coated plate at 6000 cells/cm^2^ in the culture medium. The next day, the medium was replaced with EGM2 medium (Lonza) containing 20 ng/mL BMP4 (R&D Biosystems, Minneapolis, MN), 20 ng/mL FGF8b (Peprotech, Cranbury, NJ), 1.5 μM A83-01 (TGF-β signal (TGF-β type I receptor) inhibitor; Fujifilm), and 1 mM 8-bromo-cyclic AMP (Cayman Chemical, Ann Arbor, MI). The cells were cultured for 10 days, and the medium was changed at least once every 2 days.

### 2.4 Real-time PCR

Total RNA was isolated from the cells using TRIzol™ Reagent (Thermo Fisher Scientific, Waltham, MA) according to the manufacturer's instructions. cDNA was synthesized from 500 ng of total RNA using a High-Capacity RNA-to-cDNA™ Kit (Thermo Fisher Scientific). Real-time RT-PCR was performed with the Fast SYBR™ Green Master Mix (Thermo Fisher Scientific) using a StepOnePlus real-time PCR system (Thermo Fisher Scientific). Relative quantitation of the target mRNA levels was performed using the 2^-(ΔΔ threshold cycle)^ (2^−ΔΔCT^) method. Values were normalized to those of the housekeeping gene, peptidylprolyl isomerase A (*PPIA*). The sequences of real-time PCR primers used in this study are presented in Supplementary Table S1.

### 2.5 Immunocytochemistry

The cells were fixed with methanol for 5 min at −20 °C. The cells were then treated with 0.1% Tween in PBS (phosphate-buffered saline) for 10 min at room temperature and then blocked with 10% FBS/2% bovine serum albumin, and 0.1% Tween-20 in PBS. The cells were incubated with the primary antibody (listed in able S2) at 4 °C overnight, and, finally, with the secondary antibody (listed in Table S3) at room temperature for 1 h. The cells were then stained with 4′,6-diamidino-2-phenylindole (DAPI) for 10 min and observed under a fluorescence microscope BZ-X710 (Keyence, Osaka, Japan).

### 2.6 Flow cytometry

Cell suspensions of BM-MSC derivatives were fixed in 2% paraformaldehyde for 10 min, permeabilized with 1 × permeabilization buffer (Thermo Fisher Scientific), and incubated with primary antibodies (listed in Table S4) at 4 °C for 30 min, followed by secondary antibodies (listed in Table S5) at 4 °C for 30 min, if necessary. Flow cytometry was performed using the Spectral Analyzer SA3800 (SONY, Tokyo, Japan).

### 2.7 Matrigel tube-formation assay

The cells were plated on Matrigel (Corning, Corning, NY)-coated plates to induce tube formation. Two days later, the cells were observed using the EVOS XL core imaging system (Thermo Fisher Scientific).

### 2.8 Cellular uptake of low-density lipoproteins (LDLs)

The cells were cultured in a medium containing 10 μg/mL Alexa Fluor 488-labeled acetylated LDL (acLDL; Thermo Fisher Scientific) for 3 h. The cells were then stained with DAPI for 10 min after washing with PBS and observed under a fluorescence microscope BZ-X710 (Keyence).

### 2.9 Cellular uptake of hyaluronic acid (HA)

The cells were cultured in a medium containing 100 μg/mL fluoresceinamine-labeled HA (PG Research, Tokyo, Japan) for 3 h. The cells were then stained with DAPI for 10 min after washing with PBS and observed under a fluorescence microscope BZ-X710 (Keyence).

### 2.10 Cellular uptake of IgG

The cells were cultured in a medium containing 100 μg/mL IgG conjugated with Alexa Fluor-488 (donkey anti-rabbit IgG: ab150073; Abcam, Cambridge, UK) for 2 h. The cells were then stained with DAPI for 10 min after washing with PBS and then observed under a fluorescence microscope BZ-X710 (Keyence).

## 3 Result

### 3.1 Treatment with BMP4, FGF8b, TGF-β signal inhibitor, and cAMP promotes LSEC differentiation

**Fig. 1:**
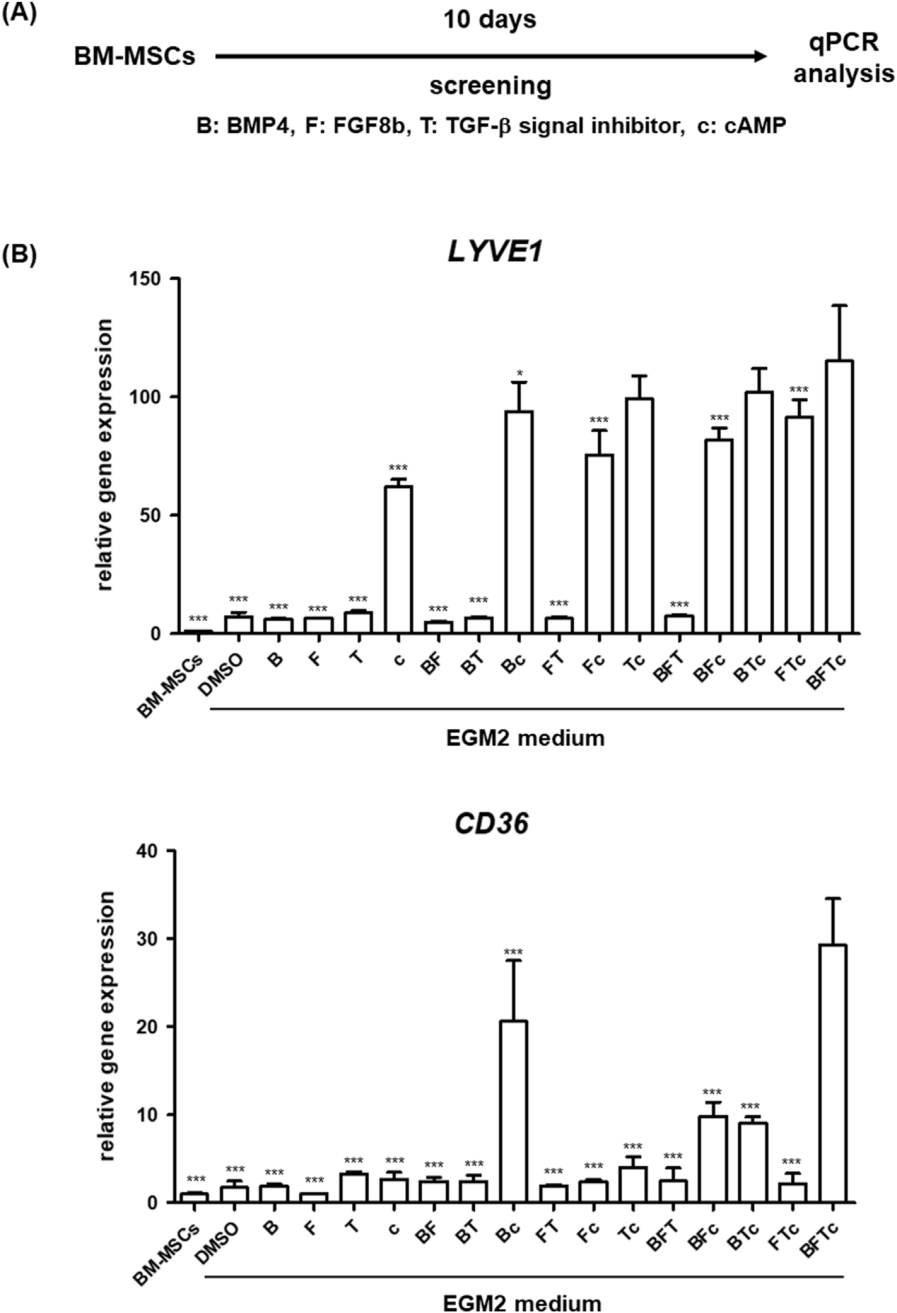
Screening of cytokines and small molecules for LSEC differentiation. (A) The schematic representation of BM-MSC differentiation. (B) qPCR analysis of the expression levels of *LYVE1* and *CD36* in BM-MSC-derived cells. On the y-axis, the expression levels are shown as a relative value to those of BM-MSCs. All data are presented as mean ± SD (n = 3). Significant differences were evaluated using a one-way ANOVA followed by Dunnett's post-hoc test (∗*p* < 0.05, ∗∗∗*p* < 0.001 compared with BFTc [BMP4, FGF8b, TGF-β inhibitor, and cAMP]). Abbreviations: B; BMP4, F; FGF8b, T; TGF-β signal inhibitor, c; cAMP.

**Fig. 2:**
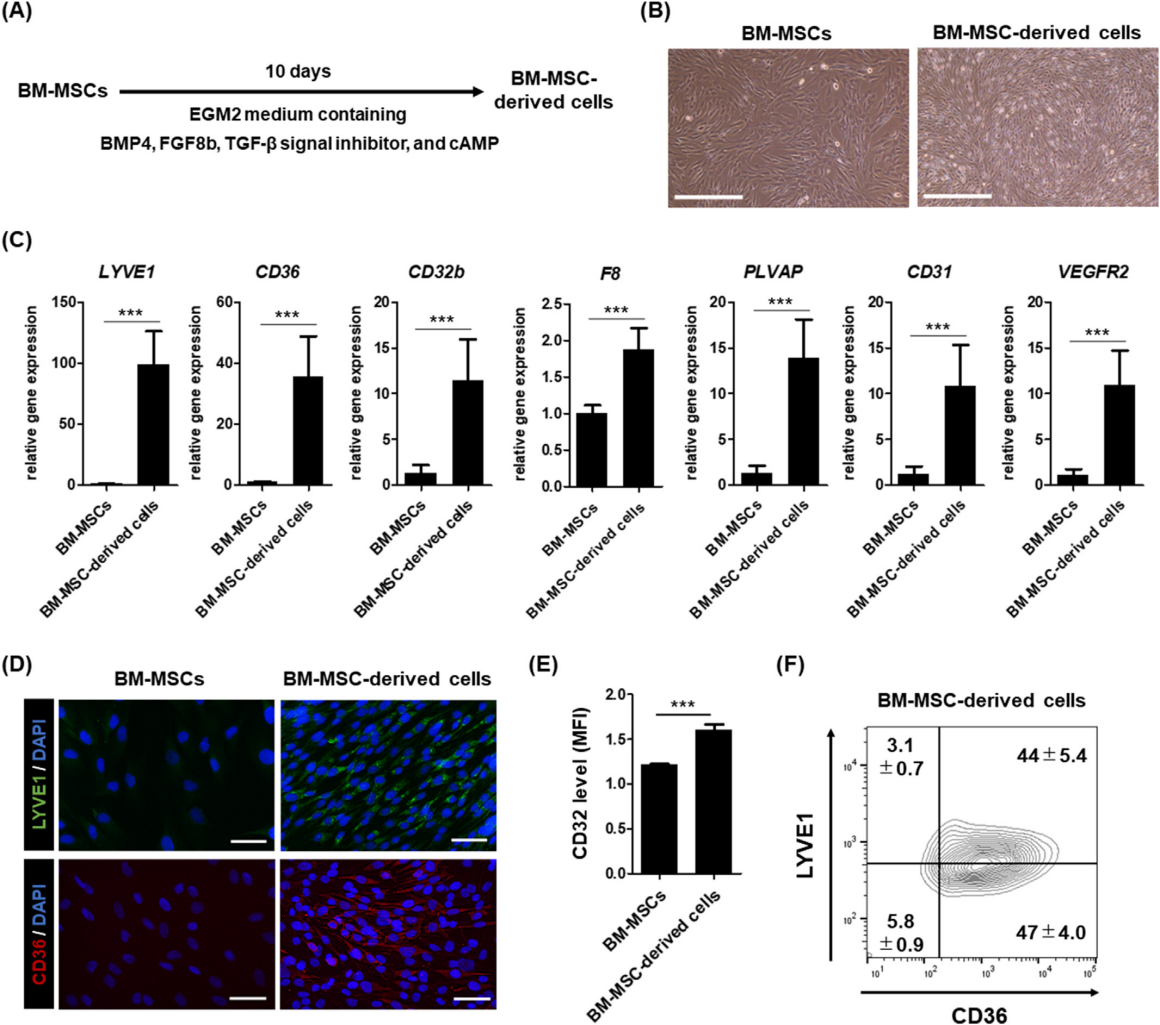
Analysis of the expression of LSEC-related markers. (A) The schematic representation of BM-MSC differentiation to LSEC-like cells. (B) Brightfield images of the BM-MSCs and BM-MSC-derived cells. Scale bar = 500 μm. (C) qPCR analysis of gene expression of *LYVE1*, *CD36*, *CD32b*, *F8*, *PLVAP*, *CD31*, and *VEGFR2* in the BM-MSCs and BM-MSC-derived cells. On the y-axis, the expression levels are shown as a relative value to those of BM-MSCs. Data are presented as mean ± SD (n = 6, sum of two independent experiments, n = 3 for each experiment). (D) Immunocytochemistry analysis of LYVE1 (green) and CD36 (red) in the BM-MSCs and BM-MSC-derived cells. The nuclei were counterstained with DAPI (blue). Scale bar = 50 μm. (E) The expression level of CD32 in the BM-MSCs and BM-MSC-derived cells was measured using flow cytometry analysis. The values of mean fluorescence intensity (MFI) of CD32 expression were normalized by the MFI of isotype control. Data are presented as mean ± SD (n = 3). Significant differences were evaluated using an unpaired two-tailed Student's *t*-test (∗∗∗*p* < 0.001). (F) The percentage of LYVE1- or CD36-positive cells in the BM-MSC-derived cells was measured using flow cytometry analysis.

To establish a protocol for the differentiation of human BM-MSCs into LSECs, we used immortalized human BM-MSCs, UE7T-13 cells [13]. We examined and screened the effects of several cytokines and chemical compounds that are known to regulate signals that play important roles in liver development [14,15] or EC and LSEC differentiation [16,17]. Specifically, we focused on BMP4, FGF8b, A83-01 (TGF-β signal inhibitor), and cAMP and evaluated combinations of the four molecules for the differentiation of BM-MSCs into LSECs by culturing the cells for 10 days (Fig. 1A). The gene expression levels of LSEC-related markers (lymphatic vessel endothelial hyaluronan receptor 1 [*LYVE1*] and *CD36*) [18] were significantly and most upregulated in the cells when they were cultured in EGM2 medium containing all four molecules (Fig. 1B). Therefore, we employed this differentiation protocol in the subsequent experiments (Fig. 2A). We then performed detailed analyses of the differentiated BM-MSC-derived cells. Brightfield images of the BM-MSCs and BM-MSC-derived cells are shown in Fig. 2B. We observed that the cell morphology was altered with the induction of differentiation. Regarding gene expression levels, the expression of LSEC and EC-related genes (*LYVE1*, *CD36*, *CD32b*, which is also known as FCγRIIb, coagulation factor VIII [*F8*], plasmalemma vesicle-associated protein [*PLVAP*], *CD31*, and vascular endothelial growth factor receptor 2 [*VEGFR2*]) [7,[18], [19], [20]] in the differentiated BM-MSC-derived cells was significantly higher than that in the BM-MSCs (Fig. 2C), whereas their expression was lower than that in the adult human liver (Fig. S1). The expression of several genes, such as *CD32b*, *F8*, and *CD36*, in the differentiated BM-MSC-derived cells, was higher than that in human cultured LSECs (Fig. S1). In contrast, the gene expression of the lymphatic EC (LEC) marker (podoplanin [*PDPN*] [19]) in the BM-MSC-derived cells was lower than that in the BM-MSCs (Fig. S2). Moreover, the protein expression of LSEC markers (LYVE1 and CD36) was detected in the differentiated BM-MSC-derived cells using fluorescent immunostaining (Fig. 2D). The protein expression of CD32 in the BM-MSC-derived cells was significantly higher than that in BM-MSCs (Fig. 2E). To evaluate the differentiation efficiency of LSECs from BM-MSCs, we measured the percentage of LYVE1- or CD36-positive cells among the differentiated BM-MSC-derived cells using flow cytometry. The percentage of LYVE1- and CD36-double positive BM-MSC-derived cells was approximately 44% (Fig. 2F). These results suggest that human BM-MSCs could differentiate into LSEC-like cells when cultured in EGM2 medium containing BMP4, FGF8b, A83-01, and cAMP for 10 days.

### 3.2 Differentiated BM-MSC-derived cells exhibit EC-related functions

**Fig. 3:**
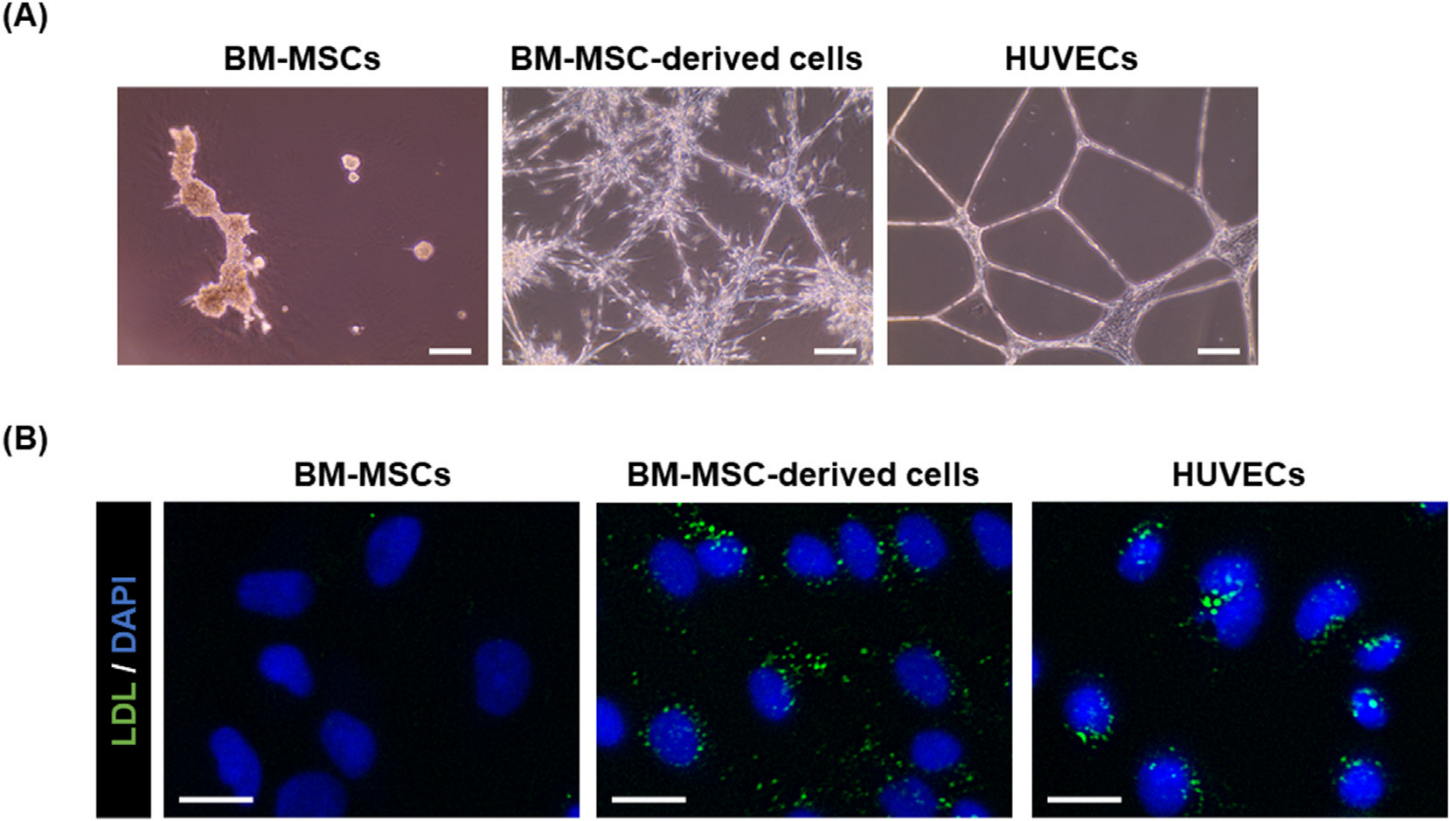
Evaluation of endothelial cell-related functions. (A) Tube-formation assay of the BM-MSCs, BM-MSC-derived cells, and HUVECs. Scale bar = 200 μm. (B) acLDL-uptake assay. BM-MSCs, BM-MSC-derived cells, and HUVECs were cultured in a medium containing Alexa Fluor 488-conjugated acLDL to measure their ability to take up acLDL. Scale bar = 20 μm. Abbreviation: LDL, low-density lipoprotein.

As ECs, including LSECs, have the ability to form tube structures on Matrigel in vitro [21], we evaluated the tube-forming ability of the differentiated BM-MSC-derived cells. Undifferentiated BM-MSCs, differentiated BM-MSC-derived cells, and HUVECs (as an EC control) were seeded onto Matrigel-coated plates, cultured for two days, and observed under a microscope. The differentiated BM-MSC-derived cells and HUVECs formed tube structures in vitro, whereas undifferentiated BM-MSCs did not (Fig. 3A). However, the differentiated BM-MSC-derived cells formed some frizzled tube-like structures. In addition, because ECs, including LSECs, have the ability to take up LDLs [22,23], we examined the LDL-uptake ability of undifferentiated BM-MSCs, differentiated BM-MSC-derived cells, and HUVECs. The cells were treated with Alexa Fluor 488-labeled acLDL for 3 h and then observed under a fluorescence microscope. The differentiated BM-MSC-derived cells and HUVECs were observed to uptake acLDL; however, undifferentiated BM-MSCs did not (Fig. 3B). These results indicate that the differentiated BM-MSC-derived cells possessed EC-specific functional properties.

### 3.3 Differentiated BM-MSC-derived cells exhibit LSEC-related functions

**Fig. 4:**
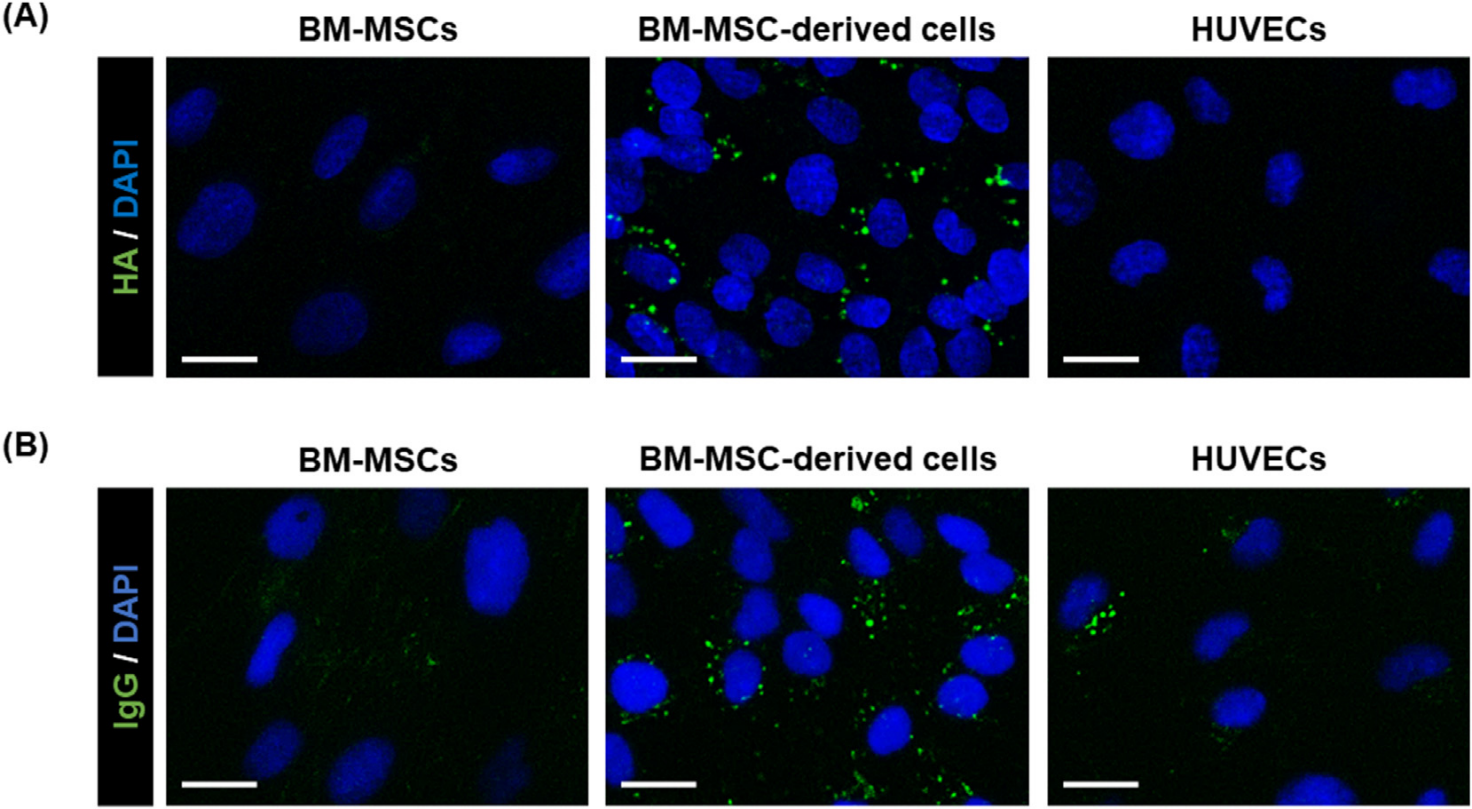
Evaluation of LSEC-related functions. (A) HA-uptake assay. BM-MSCs, BM-MSC-derived cells, and HUVECs were cultured in a medium containing fluoresceinamine-conjugated HA to measure their ability to take up HA. (B) IgG-uptake assay. BM-MSCs, BM-MSC-derived cells, and HUVECs were cultured in a medium containing Alexa Fluor 488-conjugated IgG to measure their ability to endocytose IgG. Scale bar = 20 μm. Abbreviation: HA, hyaluronic acid.

As LSECs can take up HA [24,25], we examined the HA-uptake ability of the differentiated BM-MSC-derived cells. Undifferentiated BM-MSC, differentiated BM-MSC-derived cells, and HUVECs were treated with fluoresceinamine-labeled HA for 3 h and then observed under a fluorescence microscope. The differentiated BM-MSC-derived cells were observed to take up HA; however, undifferentiated BM-MSCs and HUVECs did not (Fig. 4A). Nevertheless, the gene expression of CD44, a HA receptor, was comparable between undifferentiated and differentiated cells (Fig. S3). As LSECs have an ability to take up immune complexes by endocytosis [[26], [27], [28]], we examined the ability of these cells to take up IgG. Undifferentiated BM-MSC, differentiated BM-MSC-derived cells, and HUVECs were treated with Alexa Fluor 488-labeled IgG for 2 h and then observed under a fluorescence microscope. The differentiated BM-MSC-derived cells were observed to take up IgG, whereas undifferentiated BM-MSC and HUVECs exhibited weak uptake of the antibodies (Fig. 4B). Overall, these results suggest that the differentiated BM-MSC-derived cells acquired LSEC-specific functions.

### 3.4 Primary human BM-MSCs could also differentiate into functional LSEC-like cells

**Fig. 5:**
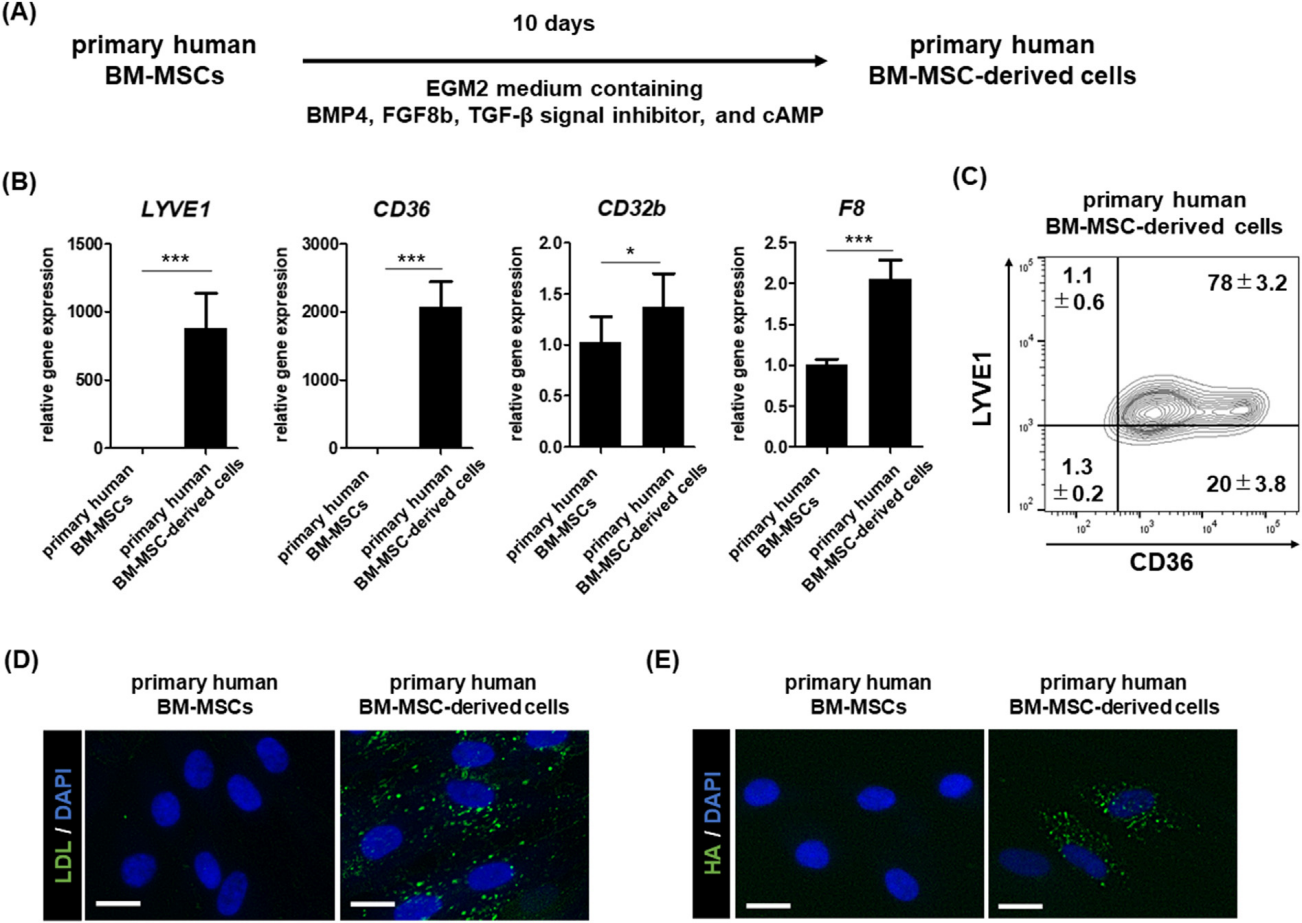
Evaluation of the differentiation capacity of primary human BM-MSCs into LSEC-like cells (A) The schematic representation of the differentiation of primary human BM-MSCs into LSEC-like cells. (B) qPCR analysis of gene expression of *LYVE1*, *CD36*, *CD32b*, and *F8* in primary human BM-MSCs and primary human BM-MSC-derived cells. On the y-axis, the expression levels are shown as a relative value to those of primary human BM-MSCs. Data are presented as mean ± SD (n ≥ 8, sum of two independent experiments, n ≥ 3 for each experiment.). Significant differences were evaluated using an unpaired two-tailed Student's *t*-test (∗*p* < 0.05, ∗∗∗*p* < 0.001). (C) The percentage of LYVE1- or CD36-positive cells in the primary human BM-MSC-derived cells was measured using flow cytometry analysis. Data are presented as mean ± SD (n = 3). (D) acLDL-uptake assay. BM-MSCs, BM-MSC-derived cells, and HUVECs were cultured in a medium containing Alexa Fluor 488-conjugated acLDL to measure their ability to take up acLDL. Scale bar = 20 μm. (E) HA-uptake assay. Primary human BM-MSCs and primary human BM-MSC-derived cells were cultured in a medium containing fluoresceinamine-conjugated HA to measure their ability to take up HA. Scale bar = 20 μm. Abbreviation: LDL, low-density lipoprotein; HA, hyaluronic acid.

We attempted to differentiate primary human BM-MSCs, not immortalized BM-MSCs (UE7T-13), into LSEC-like cells (Fig. 5A). The expression of LSEC-specific genes was upregulated by LSEC differentiation (Fig. 5B). In addition, the percentage of LYVE1- and CD36-double positive primary human BM-MSC-derived cells was approximately 78% (Fig. 5C). Furthermore, the differentiated primary human BM-MSC-derived cells exhibited LSEC-related functions (acLDL- and HA-uptake capacities) (Fig. 5D and 5E). These results indicate that primary human BM-MSC could also differentiate into LSEC-like cells when cultured in EGM2 medium containing BMP4, FGF8b, A83-01, and cAMP for 10 days.

### 3.5 Human UCB-MSCs also induce LSEC marker gene expression

To evaluate the differentiation capacity of MSCs from different tissue origins, we cultured human UCB-MSCs (UCB408E7-TERT34 cells) in EGM2 medium containing BMP4, FGF8b, A83-01, and cAMP for 10 days (Fig. S4A). The gene expression of LSEC markers (*LYVE1*, *CD36*, *CD32b*, and *F8*) in UCB-MSC-derived cells was significantly higher than that in undifferentiated UCB-MSCs (Fig. S4B).

## 4 Discussion

In this study, we developed a method for differentiating LSECs from human MSCs. We found that human BM-MSCs acquired LSEC-specific properties when cultured in EGM2 medium containing BMP4, FGF8b, A83-01, and cAMP for 10 days.

After the screening (Fig. 1), we clarified that not only the suppression of the TGF-β signaling pathway [17,22,29] and cAMP [16,30,31], which play important roles in the differentiation of vascular ECs and LSECs, but also the FGF and BMP signaling pathways, which play important roles in liver bud formation, is necessary for the efficient differentiation of human BM-MSCs into LSEC-like cells. As liver bud formation is promoted by preceding angiogenesis [32], signals that are important in the early stages of liver bud formation are speculated to also be involved in LSEC differentiation.

PDPN is a marker for LECs, whereas LYVE1 is a marker of LECs as well as LSECs [23]. The expression of *LYVE1* was significantly upregulated, whereas that of *PDPN* was not increased (Fig. S2). These results indicate that BM-MSCs did not differentiate into lymphatic vessel ECs by induction of differentiation.

As shown in Fig. 3, the BM-MSC-derived cells acquired tube-formation and LDL-uptake abilities, similar to HUVECs, suggesting that the BM-MSC-derived cells acquire the characteristics of vascular ECs by differentiation, although the gene expression of EC markers was low. Although the differentiated BM-MSC-derived cells formed frizzled tube-like structures, this phenomenon might be attributed to contaminated non-LSEC-like cells, which were partially mixed. Furthermore, LSEC-specific functions, such as HA uptake and IgG endocytosis, were observed (Fig. 4). CD44 and LYVE1 are receptors for HA [33], and CD44 is also a marker for MSCs [34]. As there were no differences in the gene expression of CD44 between BM-MSC and BM-MSC-derived cells (Fig. S3), the differences in the expression of LYVE1 likely caused differences in the uptake capacity. CD32b is the primary Fc receptor in LSECs for the endocytosis of immune complexes [27]. We observed that the gene expression of CD32b and protein expression of CD32 in the differentiated cells was increased (Fig. 2C and E), indicating the induction of functional CD32b expression. In addition, ECs have been shown to also take up some IgG [28]. Herein, we observed some IgG uptake by the HUVECs (Fig. 4).

The method developed in this study could be used to produce LSEC-like cells in just 10 days, which is shorter than that of previously reported methods [12] for producing LSEC-like cells from MSCs. Considering the percentage of LSEC marker-positive cells in MSC-derived cells (Fig. 2, Fig. 5C), the differentiation efficiency of our method was determined to be 44% or more, which is higher than that for previously reported methods [12]. Furthermore, our method is simpler because it involves single-step induction of differentiation, whereas previously reported methods involve multiple steps [12]. Therefore, our method can not only yield functional human LSEC-like cells but also overcome some limitations of the previously reported LSEC-like cell-induction methods. In addition, as different donor-derived MSCs could differentiate into functional LSEC-like cells, our method might be universally applied for LSEC differentiation from MSCs. Although MSCs are known to show different differentiation directions depending on the tissue of origin [35], our results show that human UCB-MSCs might be differentiated into LSEC-like cells as well as human BM-MSCs using our method (Fig. S4).

Cells differentiated from human BM-MSCs using our method acquired the properties of LSECs. However, the expression of various LSEC-related genes was lower than that in the adult liver, albeit higher than that in human cultured LSECs (Fig. S1). Human LSECs used in this study were commercially purchased. The cells were cultured in vitro once and then frozen; therefore, they might have lost their ability to strongly express LSEC-specific markers [10,11]. This might be the cause for the lower expression of LSEC-specific genes in human LSEC samples than that in the adult liver sample observed in this study (Fig. S1). Hence, although the BM-MSC-derived cells obtained by differentiation induction are functional, they are likely to be immature LSECs. In this study, we focused on early hepatic developmental signals, such as FGF and BMP. Therefore, examining whether further maturation is possible by focusing on the signaling pathways involved in late hepatic development or on interactions with other constituent cells of the liver is necessary.

## 5 Conclusions

We successfully induced the differentiation of functional LSECs from human BM-MSCs. We hope that our findings and differentiation-induced cells will promote further research on LSECs and the development of medical technologies for liver diseases.

## Author contribution

Conceptualization, S.M.; Investigation, S.M., Y.O., C.H., Y.T., and A.S.; Formal Analysis, S.M.; Data Curation, S.M.; Writing – Original Draft, S.M.; Writing – Review & Editing, S.M., M.S., and K.T; Funding Acquisition, S.M., M.S., and K.T.

## Funding

This work was supported by the 10.13039/501100001691Japan Society for the Promotion of Science (JSPS KAKENHI) [grant numbers 21K16008 and 20H00531]; and the TERUMO LIFE SCIENCE FOUNDATION, The Mother and Child Health Foundation, and Novo Nordisk Access to Insight 2021 Basic Research Grant.

## Declaration of competing interest

The authors declare the following financial interests/personal relationships which may be considered as potential competing interests: Kohei Tatsumi reports financial support was provided by Novo Nordisk. Seiji Mitani reports a relationship with Bayer that includes: funding grants. Midori Shima reports a relationship with Chugai Pharmaceutical Co., Ltd. that includes: consulting or advisory, funding grants, and speaking and lecture fees. Midori Shima reports a relationship with Takeda Pharmaceutical Co., Ltd. that includes: funding grants and speaking and lecture fees. Midori Shima reports a relationship with CSL Behring that includes: funding grants and speaking and lecture fees. Midori Shima reports a relationship with Sanofi that includes: speaking and lecture fees. Midori Shima reports a relationship with Bayer that includes: speaking and lecture fees. Midori Shima reports a relationship with Novo Nordisk that includes: speaking and lecture fees. Midori Shima reports a relationship with Pfizer that includes: speaking and lecture fees. Midori Shima reports a relationship with Fujimoto Seiyaku Corp. that includes: speaking and lecture fees. Asuka Sakata reports a relationship with CSL Behring that includes: speaking and lecture fees.
