# Supplementary material for: Generation of functional liver sinusoidal endothelial-like cells from human bone marrow-derived mesenchymal stem cells
Source: Regen Ther. 2023 Aug 1;24:274–81. doi: 10.1016/j.reth.2023.07.006 (PMC10412721; doi:10.1016/j.reth.2023.07.006)
Supplement: Multimedia component 1 [file mmc1.docx]

**Supplementary Materials**

**Title**

**Generation of functional liver sinusoidal endothelial-like cells from human bone marrow-derived mesenchymal stem cells**

**Authors**

Seiji Mitani, Yu Onodera, Chihiro Hosoda, Yoko Takabayashi, Asuka Sakata, Midori Shima, and Kohei Tatsumi


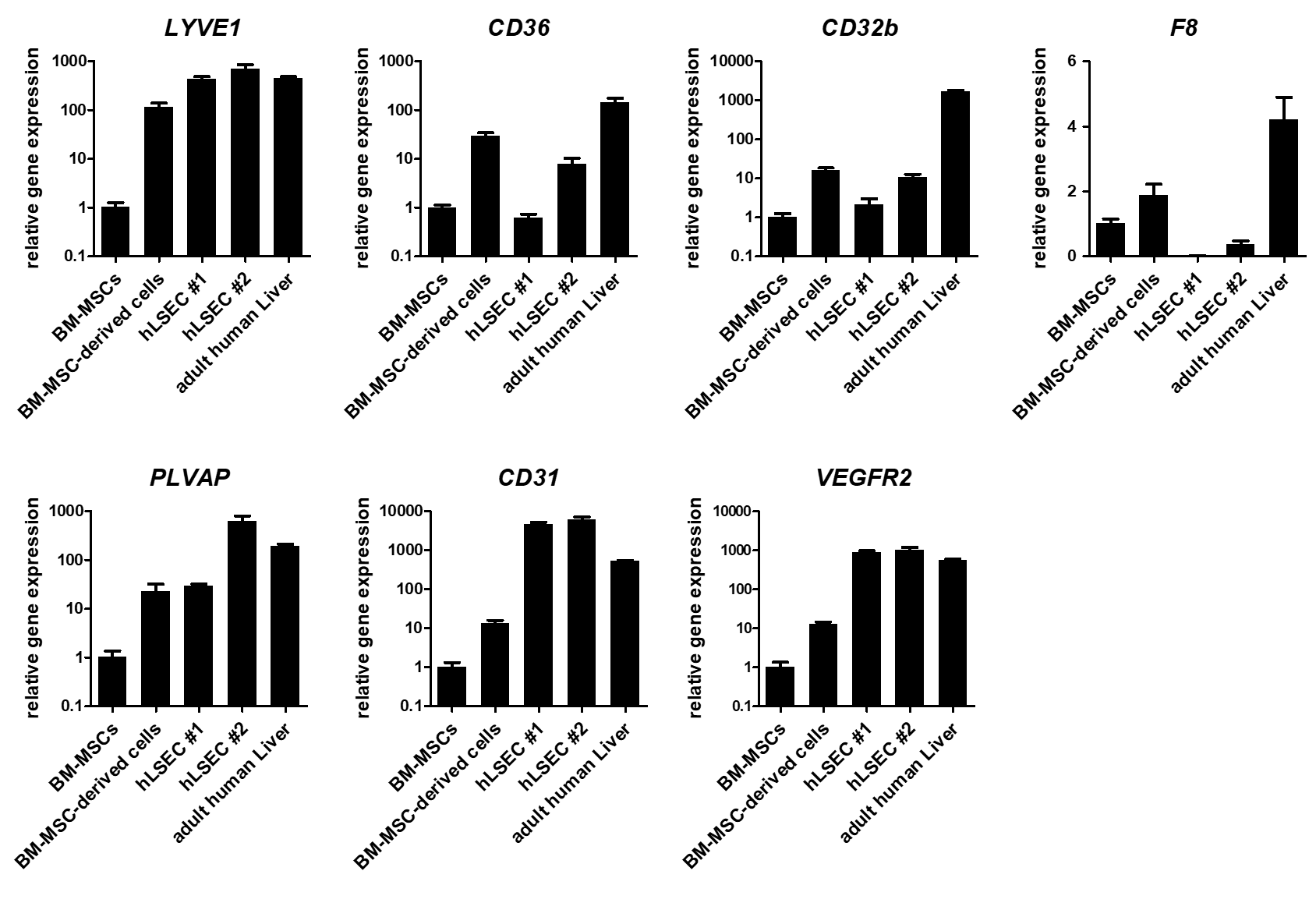


Figure S1. Analysis of the gene expression profiles in cells and tissue.

qPCR analysis of gene expression of *LYVE1*, *CD36*, *CD32b*, *F8*, *PLVAP*, *CD31*, and *VEGFR2* in the BM-MSCs, BM-MSC-derived cells, primary human LSECs, and adult human liver. On the y-axis, the expression levels are shown as a relative value to those of BM-MSCs. All data are presented as mean ± SD (n = 3).


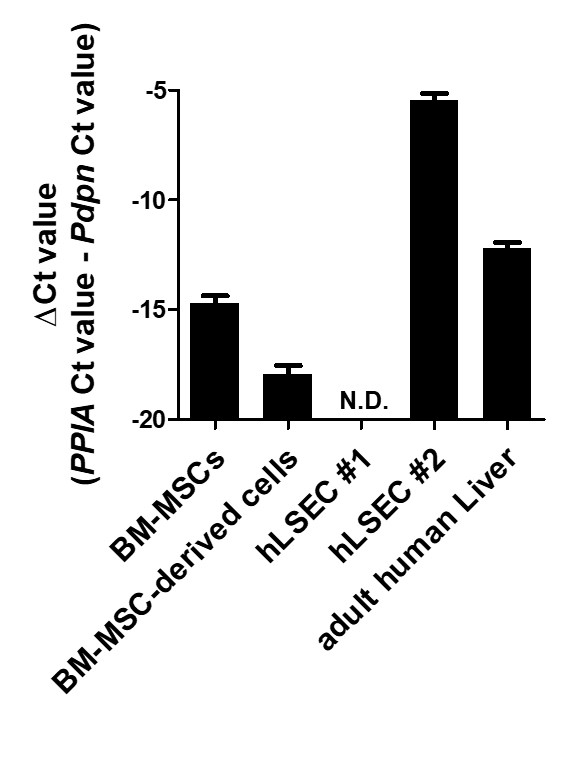


Figure S2. Analysis of the lymphatic endothelial cell marker expression profile.

qPCR analysis of gene expression of *PDPN* (podoplanin) in the BM-MSCs, BM-MSC-derived cells, primary human LSECs, and adult human liver. Data are presented as mean ± SD (n = 3).


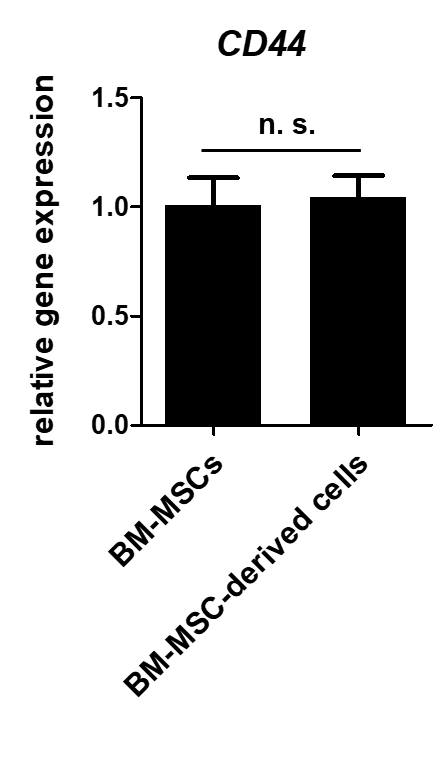


Figure S3. Analysis of hyaluronic acid receptor expression.

qPCR analysis of gene expression of *CD44* in the BM-MSCs and BM-MSC-derived cells. On the y-axis, the expression levels are shown as a relative value to those of BM-MSCs. Data are presented as mean ± SD (n = 3). Significant differences were evaluated using an unpaired two-tailed Student’s *t*-test. Abbreviations: n. s., not significant.


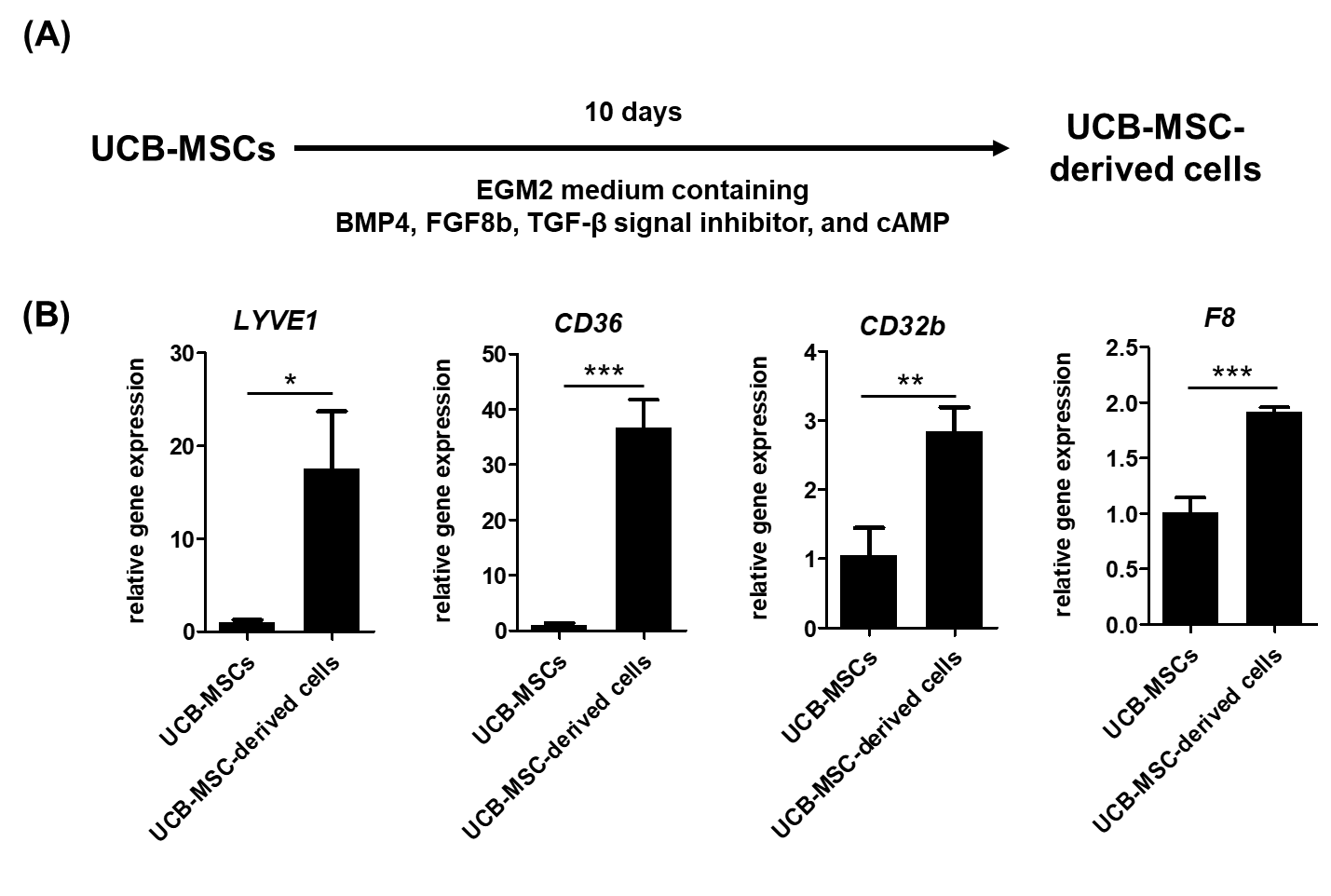


Figure S4. Evaluation of the LSEC differentiation capacity of the umbilical cord blood-derived MSCs (UCB-MSCs).

(A) The schematic representation of UCB-MSC differentiation. (B) qPCR analysis of LSEC-specific gene expression of *LYVE1*, *CD36*, *CD32b*, and *F8* in the human UCB-MSCs and their derivatives. On the y-axis, the expression levels are shown as a relative value to those of UCB-MSCs. All data are presented as mean ± SD (n = 3). Significant differences were evaluated using an unpaired two-tailed Student’s *t*-test (**p* < 0.05, ***p* < 0.01, ****p* < 0.001).

Table S1. Sequences of the primers used for real-time PCR

| Gene symbol | Primer sequence (forward/reverse; 5’ to 3’) |
| --- | --- |
| *LYVE1* | AGGCTCTTTGCGTGCAGAA/ GGTTCGCCTTTTTGCTCACAA |
| *CD32* | AGCCAATCCCACTAATCCTGA/ GGTGCATGAGAAGTGAATAGGTG |
| *CD36* | GGCTGTGACCGGAACTGTG/ AGGTCTCCAACTGGCATTAGAA |
| *F8* | GCATTCGCAGCACTCTTCG/ GAGGTGAAGTCGAGCTTTTGAA |
| *PLVAP* | GCTGCTGGTATTACCTGCG/ GCCATAGACCATGAAGAGCAC |
| *CD31* | AACAGTGTTGACATGAAGAGCC/ TGTAAAACAGCACGTCATCCTT |
| *VEGFR2* | GGCCCAATAATCAGAGTGGCA/ CCAGTGTCATTTCCGATCACTTT |
| *podoplanin* | AACCAGCGAAGACCGCTATAA/ CGAATGCCTGTTACACTGTTGA |
| *CD44* | CTGCCGCTTTGCAGGTGTA/ CATTGTGGGCAAGGTGCTATT |
| *PPIA* | CCCACCGTGTTCTTCGACATT/ GGACCCGTATGCTTTAGGATGA |

Abbreviations: LYVE1; lymphatic vessel endothelial hyaluronan receptor 1, F8; coagulation factor VIII, PLVAP; plasmalemma vesicle associated protein, VEGFR2; vascular endothelial growth factor receptor 2, PPIA; peptidylprolyl isomerase A.

Table S2. The primary antibodies used for immunocytochemistry

| Antigen | Type | Company | Catalogue number |
| --- | --- | --- | --- |
| Human CD36 | Rabbit | Novous Biologicals | NB400-144SS |
| Human LYVE1 | Rabbit | abcam | ab33682 |

Table S3. The secondly antibodies used for immunocytochemistry

| Antigen | Label | Company | Catalogue number |
| --- | --- | --- | --- |
| Rabbit IgG | Alexa Fluor 488 | abcam | ab150073 |
| Rabbit IgG | Alexa Fluor 647 | abcam | ab150075 |

Table S4. The primary antibodies used for flow cytometry analysis

| Antigen | Type | Company | Catalogue number |
| --- | --- | --- | --- |
| Human CD36 | Mouse | abcam | ab17044 |
| Human LYVE1 | Rabbit | abcam | ab33682 |
| Human CD32 | Mouse | Biolegend | 303204 |

Table S5. The secondly antibodies used for flow cytometry analysis

| Antigen | Label | Company | Catalogue number |
| --- | --- | --- | --- |
| Mouse IgG | Alexa Fluor 647 | abcam | ab150115 |
| Rabbit IgG | Alexa Fluor 488 | abcam | ab150073 |
